# Tuberculosis: insights into latency, treatment strategies, and the potential roles of metronidazole

**DOI:** 10.3389/ftubr.2023.1230713

**Published:** 2023-08-23

**Authors:** Harald G. Wiker

**Affiliations:** ^1^Department of Clinical Science, University of Bergen, Bergen, Norway; ^2^Department of Microbiology, Haukeland University Hospital, Bergen, Norway

**Keywords:** dormancy, drug-resistance, latency, metronidazole, microbiome, *Mycobacterium tuberculosis*, tuberculosis

## Abstract

Recent studies challenge the traditional belief that latent tuberculosis is a lifelong condition. Analysis of tuberculosis decline in low endemic areas suggests that latency is diminishing, and a significant proportion of individuals likely clear the infection each year. The risk of developing active tuberculosis diminishes over time, highlighting the importance of early intervention for optimal treatment outcomes. The global estimate of latent tuberculosis infection has been revised to approximately 23% of the global population. Latent bacilli undergo mutation and proliferation, contrary to the previous notion of dormancy. Treatment regimens for tuberculosis, particularly multidrug-resistant strains, have improved with the BPaLM regimen. However, current treatment approaches for drug sensitive tuberculosis do not specifically target dormant bacilli, necessitating the development of better protocols. Metronidazole shows potential in killing non-proliferating *Mycobacterium tuberculosis*, but its clinical effectiveness remains uncertain. Adverse effects and drug interactions of metronidazole should be considered. Alternative treatment options and the role of lung flora in tuberculosis therapy require further investigation. The suitability of metronidazole for this purpose remains open.

## 1. Introduction

Latent tuberculosis is commonly believed to be a lifelong condition, implying that once someone is infected with tubercle bacilli, they will carry the bacteria for the remainder of their life. However, recent studies challenge this notion. Analysis of the decline of tuberculosis in low endemic areas like Norway (1) and Sweden (2), where recent transmission is infrequent and most cases arise from reactivation of past infections, has revealed that latency is diminishing, and a significant proportion of individuals likely clear the infection each year. Although there are instances where latency can persist for several decades in certain individuals (3, 4), epidemiological data suggest that such cases are exceptional. Consequently, it has been pointed out that there is no clear distinction between primary disease and reactivation (5).

The risk of developing active tuberculosis in an infected individual is highest during the initial years after infection, but gradually diminishes over time (6). This has significant practical implications, particularly regarding the treatment of latent tuberculosis. Early intervention following an infectious episode is crucial for achieving optimal treatment outcomes. Therefore, it becomes essential to gather information regarding the timing of the probable infectious episode when obtaining the patient's medical history.

Another important implication is that the global estimate of individuals with latent tuberculosis infection has been significantly exaggerated in terms of a microbiological definition of latency. Previously, it was commonly cited that approximately one third of the world's population carried latent infection (7). However, a more recent investigation has provided revised figures, suggesting that in 2014, the actual percentage was estimated to be around 23% (with a range of 20.4 to 26.4%) of the global population (8). This updated assessment is believed to be a more accurate approximation, bringing us closer to the true prevalence of latent tuberculosis infection worldwide.

## 2. Dynamics of latency and dormancy

Previously, the concept of latency in tuberculosis suggested a state of dormancy, where individuals harbored tubercle bacilli that did not actively multiply. However, recent findings from whole genome sequencing have challenged this understanding. It has been revealed that latent bacilli actually undergo mutation at a rate comparable to, or slightly lower than, the mutation rate observed in active tuberculosis cases (9–12). These results have significant implications, indicating that there is substantial proliferation of tubercle bacilli during the latent stage, contrary to the previous notion of dormancy.

The phenomenon of dormancy, characterized by the non-replication of *M. tuberculosis*, is an unquestionable occurrence that has been demonstrated both experimentally and *in vivo*. *In vitro* experiments have successfully induced dormancy by gradually exposing tubercle bacilli to reduced oxygen levels (13). This controlled environment enables the bacilli to adapt their metabolism and enter a non-proliferative state. Moreover, evidence from various animal models has shown the presence of hypoxic conditions within tuberculous granulomas, further supporting the existence of dormancy *in vivo* (14). These findings underscore the presence of dormancy as a significant aspect of *M. tuberculosis* infection, both within laboratory settings and in natural disease conditions.

However, the precise mechanisms underlying latency in humans remain largely unknown. Several possibilities exist regarding the nature of latency: it could involve prolonged periods of dormancy interspersed with sporadic episodes of bacilli proliferation, or it could entail continuous bacilli proliferation that is effectively controlled by immune-mediated killing. Another intriguing hypothesis suggests the presence of two distinct pools of bacilli within the body: one in a dormant state and the other actively proliferating. The transition between these states is likely influenced by factors that regulate bacilli containment and immune response. Ultimately, the outcome of latency can vary, leading to either complete eradication of the bacilli and resolution of the infection or reactivation, characterized by clinical symptoms and the development of overt disease. Further research is needed to elucidate the precise dynamics and factors involved in these different scenarios of latency in order to better understand tuberculosis infection in humans.

Tubercle bacilli are aerobic organisms that rely on oxygen for their replication and exhibit optimal growth conditions in the lung apices, where oxygen tension is highest. However, intriguingly, these bacilli have also demonstrated the ability to survive in environments with reduced oxygen levels if the transition occurs gradually enough. The presence of dormant bacilli during active disease is an important question that merits exploration. Intuitively, many would assume the existence of such dormant bacilli within the host during active tuberculosis.

## 3. Treatment strategies for tuberculosis

The treatment of multidrug-resistant tuberculosis has witnessed significant advancements with the introduction of the BPaLM regimen, which has shown remarkable efficacy. This treatment approach combines Bedaquiline, Pretomanid, Linezolid, and Moxifloxacin as oral medications, administered for a period of only 6 months (15). Notably, this drug combination is designed to target both proliferating and non-proliferating tubercle bacilli, addressing the diverse states of the bacteria during infection. Such an approach ensures a comprehensive therapeutic strategy that effectively tackles the entire spectrum of tubercle bacilli populations.

The current recommended treatment regimens for drug-sensitive tuberculosis or latent tuberculosis do not specifically target dormant bacilli, highlighting the need for further improvement. To effectively address the dormant phase of the infection, it is crucial to develop treatment protocols that incorporate drugs capable of eliminating dormant bacilli. Enhancing the treatment regimens for drug-sensitive tuberculosis or latent tuberculosis by introducing medications specifically designed to target and eradicate dormant bacilli would be a significant step toward more comprehensive and successful management of these conditions.

Metronidazole is a widely used and safe drug typically employed for the treatment of anaerobic and parasitic infections. Its efficacy relies on activation by microbial reductases. In the case of *M. tuberculosis*, several enzymes encoded by the bacterium are potential candidates for facilitating this activation process (16, 17). Experimental evidence from both *in vitro* and *in vivo* studies has demonstrated that metronidazole can effectively eliminate non-proliferating *M. tuberculosis*. However, it is important to note that some investigations have raised doubts about the drug's efficacy *in vivo*, casting uncertainty on its potential applicability for treating tuberculosis patients. Further research is needed to ascertain the true clinical effectiveness of metronidazole as a therapeutic option for tuberculosis treatment.

The effect of metronidazole on oxygen-starved bacilli was first demonstrated in the Wayne model, marking a significant milestone in understanding its impact (18). The study revealed that metronidazole exhibited a notable effect, and when combined with rifampin or isoniazid, an additive effect was observed. Subsequent investigations have consistently confirmed the efficacy of metronidazole on dormant bacilli *in vitro* (16, 17, 19). While multiple studies have shown the potential of metronidazole, it is crucial to note that only one study has reported a sterilizing effect on dormant bacilli. This particular study utilized a combination of 8 mg/L metronidazole and 1 mg/L rifampin on 26-day-old dormant cultures (17). These findings emphasize the importance of carefully adjusting the experimental conditions to accurately assess the potential of metronidazole. It is worth noting that such adjustments pose greater challenges when working with animal models, adding further complexity to the evaluation process.

## 4. Animal models for tuberculosis research

The selection of an appropriate animal model holds significant importance when studying tuberculosis. While mouse models are widely used due to their convenience, it is crucial to acknowledge that certain pathological parameters in mice differ from those observed in human tuberculosis. In this regard, many researchers prefer the guinea pig model (20) as guinea pigs are prone to developing active disease and exhibit the formation of characteristic hypoxic granulomas with necrosis and mineralization, which more closely resemble human tuberculosis pathology.

The findings from animal models of tuberculosis regarding the effects of metronidazole are not as straightforward as the findings of *in vitro* studies. In the context of active disease, two studies investigating the use of metronidazole alone or in combination with other antibiotics did not demonstrate any significant effect (20, 21). During active infection, the bulk of the bacillary burden is likely represented by proliferating bacilli, with dormant bacilli being less abundant. Given this scenario, it becomes challenging to observe the efficacy of metronidazole as a monotherapy and even harder to demonstrate an additive effect when combined with isoniazid or rifampin.

In animal models of chronic or latent infection, three studies reported positive effects with metronidazole used as monotherapy (14, 21, 22), while one study did not report any effect (23). Combination therapies involving metronidazole with isoniazid or rifampin were found to be effective in a primate model for latent tuberculosis (22) and in a mouse model (24). However, in two studies utilizing the Cornell model (25) of murine tuberculosis, no significant effect of metronidazole was observed when used in combination with isoniazid (21) or in combination with isoniazid and pyrazinamide (19). It is worth noting that the Cornell model is particularly relevant for studying the effects of metronidazole, as it is based on reactivation induction with steroids. However, this model is highly sensitive to even a few persistent bacteria upon reactivation, necessitating a sterilizing effect to demonstrate successful treatment.

Overall, the effects of metronidazole in animal models of tuberculosis are varied, with outcomes influenced by factors such as the stage of infection, the combination of drugs used, and the specific model employed. Further research is needed to fully understand the potential of metronidazole in different contexts and to identify optimal treatment strategies.

For studies aiming to mimic human tuberculosis more closely, the primate model utilizing cynomolgus macaque monkeys is considered advantageous, despite being resource-intensive. This model exhibits similarities to human tuberculosis and has been specifically designed to promote the development of latent tuberculosis in approximately 50% of infected animals (22). Importantly, positive results have been reported in the context of using metronidazole to treat latent tuberculosis in this primate model (22, 26).

Therefore, it is vital to consider that the choice of animal model can greatly impact research outcomes. While mouse models offer convenience, the guinea pig model and primate models, such as cynomolgus macaque monkeys, provide more relevant pathological features that align with human tuberculosis, allowing for a better evaluation of potential treatments, including the use of metronidazole.

## 5. Adverse effects of metronidazole and drug interactions

Concerns regarding the adverse effects of metronidazole have been raised. In a guinea pig model of active disease, a study reported that the group of mice treated with a combination of isoniazid, rifampin, pyrazinamide, and metronidazole experienced weight loss and had to be euthanized prematurely (20). In contrast, the control group receiving the three drugs without metronidazole did not encounter this issue. Furthermore, a study investigating the efficacy and safety of metronidazole in patients with multidrug-resistant tuberculosis (27) had to be terminated prematurely due to the development of peripheral neuropathies in the group receiving metronidazole in combination with second-line drugs for tuberculosis treatment. It is worth noting that the publication did not provide details regarding the specific drug combinations, which limits further in-depth analysis (27).

These adverse effects observed in both animal models and human studies suggest that caution should be exercised when considering the use of metronidazole in combination with certain anti-tuberculous drugs. The specific drug interactions and combinations may play a role in determining the occurrence of these side effects. Further research is required to better understand the potential risks associated with metronidazole and to identify appropriate strategies to mitigate these adverse effects, ensuring the safety and efficacy of tuberculosis treatment.

## 6. Considering the lung microbiome and other bacteria in tuberculosis treatment

However, one particular single-blinded clinical study from 1989 stands out in the literature (28). The study included 137 patients with advanced lung tuberculosis who were administered isoniazid, rifampicin, and streptomycin for 12 weeks and then isoniazid and rifampicin for the continuation. Among them, 76 patients received metronidazole for the first 8 weeks, while 61 patients received a placebo. The two groups were evaluated at 4, 8, and 12 weeks. Interestingly, the group receiving metronidazole showed faster improvement based on clinical criteria, radiological criteria, and sputum quantity at 4 and 8 weeks. However, by the 12-week mark, the two groups demonstrated comparable outcomes. Notably, there was no significant difference between the groups when it came to sputum culture. The authors referred to the metronidazole treatment as adjuvant therapy (28).

It is essential to explore alternative explanations for these results, because there was no evidence of a direct effect of metronidazole on *M. tuberculosis*. Traditionally, the lungs were considered sterile until around 2010/2011 when several studies on the lung microbiome (29–31) revealed that the lungs of both healthy individuals and patients with conditions like asthma or chronic obstructive lung disease were colonized by various bacterial species, including many anaerobic bacteria such as *Prevotella, Fusobacterium, Veillonella*, and *Porphyromonas*, to name a few. Therefore, considering the presence of other bacteria in cavitary pulmonary tuberculosis and the interplay between tubercle bacilli and the endogenous bacterial lung flora becomes relevant in understanding the pathogenesis of lung tuberculosis. In this context, targeting these bacteria, at least during the initial phase of tuberculosis therapy, could potentially result in faster clinical recovery, as observed in the study by Desai et al. (28). If this is indeed the case, metronidazole may not necessarily be the optimal drug choice. Other drugs that cover the normal flora of the lungs, such as beta-lactam antibiotics, might be better suited. Additionally, conducting further studies to identify the types of bacteria that thrive in cavernous tuberculosis alongside *M. tuberculosis* would be crucial in designing the most effective treatment regimen.

## 7. Improving therapy for drug-sensitive tuberculosis

The majority of tuberculosis patients are infected with drug-sensitive *M. tuberculosis* strains. While the new BPaLM regimen has shown effectiveness against drug-resistant tuberculosis by targeting both replicating and non-replicating bacilli, the standard treatment regimen for drug-sensitive tuberculosis does not adequately address the non-replicating population of tubercle bacilli. Therefore, it is crucial to prioritize the improvement of therapy for drug-sensitive tuberculosis by specifically targeting non-replicating tubercle bacilli. Whether metronidazole is a suitable drug for this purpose or if there are alternative options that might be more effective remains an open question. Metronidazole is generally considered a safe drug; however, it is essential to carefully consider potential adverse effects due to drug interactions, as indicated by the findings of Carroll et al. (27). Notably, the study by Desai et al. (28) did not report any adverse effects associated with the combination of metronidazole, isoniazid, rifampicin, and streptomycin.

The use of isoniazid monotherapy for treating latent tuberculosis seems paradoxical, given that isoniazid primarily targets replicating tubercle bacilli. However, considering the emerging evidence indicating significant bacterial multiplication during latency (9–12), the efficacy of isoniazid against latent tuberculosis becomes less surprising. Nonetheless, the use of monotherapy, whether it is isoniazid or rifampicin, for treating latent tuberculosis remains controversial due to the potential risk of drug resistance development.

Animal studies have indicated that although bacilli replicate during latency, there are also live but dormant bacilli present (14, 22), which possess the potential to reactivate tuberculosis. Therefore, it is logical to include therapy targeting dormant bacilli. One promising approach to demonstrate the effect of nitroimidazoles is to investigate their efficacy in combination with isoniazid and rifampicin in recently infected healthy individuals and closely monitor the frequency of active disease through rigorous follow-up. The aim is to develop a short-course multi-drug therapy for latent tuberculosis. In theory, the duration of antimicrobial therapy should be determined by the bacterial burden. Given the expected low bacterial burden during latency, there is potential for shorter treatment durations for latent tuberculosis.

## 8. Discussion

Recent studies challenge the notion that latent tuberculosis is a lifelong condition. While some individuals may harbor the infection for several decades, epidemiological data suggest that latency is diminishing, and a significant proportion of individuals likely clear the infection each year. The risk of developing active tuberculosis is highest during the initial years after infection but gradually diminishes over time. The global estimate of individuals with latent tuberculosis infection has been revised downward, indicating a lower prevalence than previously believed. Recent findings also challenge the concept of dormancy during latency, suggesting that tubercle bacilli undergo mutation and proliferation during this stage. However, the precise mechanisms underlying latency in humans remain largely unknown, and further research is needed to better understand tuberculosis infection.

The treatment of multidrug-resistant tuberculosis has seen significant advancements with the introduction of the BPaLM regimen, which targets both proliferating and non-proliferating tubercle bacilli. However, current treatment regimens for drug-sensitive tuberculosis and latent tuberculosis do not specifically target dormant bacilli, highlighting the need for further improvement in treatment protocols. Metronidazole has shown promise in eliminating non-proliferating *M. tuberculosis* in experimental settings, but its efficacy *in vivo* is still debated, and adverse effects have been reported.

Alternative explanations for the efficacy of metronidazole in one study suggest that the presence of other bacteria in cavitary pulmonary tuberculosis and their interplay with tubercle bacilli may influence clinical recovery. Targeting these bacteria, potentially with alternative drugs that cover the normal lung flora, could be a more effective approach. Careful consideration of potential adverse effects and drug interactions is necessary when using metronidazole in tuberculosis treatment.

## Author contributions

The author confirms being the sole contributor of this work and has approved it for publication.
